# Continuous subcutaneous insulin infusion is associated with a reduced rate of microvascular complications

**DOI:** 10.1186/1687-9856-2015-S1-O33

**Published:** 2015-04-28

**Authors:** Bedowra Zabeen, Maria E Craig, Alison Pryke, Albert K F Chan, Yoon Hi Cho, Paul Benitez Aguirre, Stephen Hing, Kim C Donaghue

**Affiliations:** 1 Institute of Endocrinology and Diabetes, The Children’s Hospital at Westmead, Australia; 2 Ophthalmology Department, The Children’s Hospital at Westmead, Australia

## Aim

To determine whether use of continuous subcutaneous insulin infusion (CSII) is associated with lower rates of microvascular complications than use of multiple daily injections (MDI) in adolescents with type 1 diabetes from 2000-2014.

## Methods

We assessed microvascular complications in 1152 adolescents aged 12-20 years with diabetes duration ≥ 5 years. Retinopathy was detected using seven–field fundal photography, albumin excretion rate (AER) using overnight urine collections or albumin-to-creatinine ratio (ACR) and peripheral nerve function by thermal and vibration threshold.

## Results

Median age was 17 years [IQR 15-18] and median diabetes duration 9.0 [7.0-12.0] years. CSII was used by 29% and MDI 72%. CSII was associated with a lower rate of retinopathy than MDI (16% vs 22%; p=0.025) across the entire study period and in the latest time period with lower rate of AER elevation (≥ 7.5 μg/min)(26%vs 37%; p=0.012); microalbuminuria(1.3% vs 5.5%; p=0.016) and peripheral nerve abnormalities (27% vs 32% ; p 0.139) although the latter did not reach statistical significance.

In multivariable analysis, retinopathy was negatively associated with CSII Odds ratio (OR) 0.68 (95%CI:0.47-0.98) and positively with higher HbA1c OR 1.20 (1.08-1.32), older age at diagnosis 1.12 (1.02-1.22), longer diabetes duration 1.26 (1.15- 1.38) and lower height SDS 0.78 (0.67-0.91). Early elevation of AER was associated with higher HbA1c OR 1.33 (1.20-1.47), insulin dose 1.86 (1.22-2.82) and lower socioeconomic advantage 0.66 (0.46-0.94). Microalbuminuria was associated with higher insulin dose 2.64 (1.07-6.50) and HbA1c 1.34 (1.07-1.68). A peripheral nerve abnormality was negatively associated with CSII OR 0.66 (0.44-0.97), insulin dose OR 0.50 (0.26- 0.94) and positively with higher BMI SDS OR 1.31(1.06-1.63).

## Conclusion

While the benefits of CSII on glycaemic control and quality of life are recognised, this is the first study to show a beneficial association of CSII vs MDI on microvascular complications.

